# Larvicidal and Repellent Effects Against Vector Mosquitoes *Anopheles stephensi* and *Aedes aegypti* (Diptera: Culicidae) for *Alstonia scholaris* Linn R Br Flower and Prospective Characterisation of Bioactive Extract

**DOI:** 10.1155/sci5/9114956

**Published:** 2026-09-03

**Authors:** Kaneez Fatima, Shaukat Khalid, Kiran Qadeer, Huma Shareef, Hina Yasin, Hina Abrar, Saima Zahid

**Affiliations:** ^1^ Department of Pharmacognosy, Baqai Institute of Pharmaceutical Sciences, Baqai Medical University, Karachi, Pakistan, baqai.edu.pk; ^2^ Department of Pharmacognosy, Institute of Pharmaceutical Sciences, Jinnah Sindh Medical University, Karachi, Pakistan, jsmu.edu.pk; ^3^ Department of Pharmaceutical Chemistry, Institute of Pharmaceutical Sciences, Jinnah Sindh Medical University, Karachi, Pakistan, jsmu.edu.pk; ^4^ Department of Pharmacognosy, Faculty of Pharmacy, Dow University of Health Sciences, Karachi, Pakistan, duhs.edu.pk; ^5^ Department of Pharmacology, Faculty of Pharmacy, Dow University of Health Sciences, Karachi, Pakistan, duhs.edu.pk; ^6^ Department of Pharmaceutical Chemistry, Baqai Institute of Pharmaceutical Sciences, Baqai Medical University, Karachi, Pakistan, baqai.edu.pk

## Abstract

Malaria and dengue are among the most widespread vector‐borne diseases globally. This study evaluated the larvicidal and repellent characteristics of *Alstonia scholaris* (Apocynaceae) flower extracts against the *Anopheles stephensi* and *Aedes aegypti* vectors. The crude methanolic extract was sequentially fractionated into ethyl acetate, *n*‐butanol and aqueous fractions. Four different fractions were tested against *Anopheles stephensi* and *Aedes aegypti* at concentrations ranging from 78 to 1092 ppm and 625–6250 ppm, respectively. Larval mortality was observed after 24 h and analysed using probit analysis to estimate lethal concentrations (LC_50_). Repellency duration was assessed for adult *Aedes aegypti* using the various solvent extracts. The methanolic extract exhibited high efficacy against both *Anopheles stephensi* and *Aedes aegypti* larvae, with LC_50_ of 312 and 960 ppm, respectively. The ethyl acetate extract showed strong repellent activity, which is persistent up to 9 h (posttreatment) against adult *Aedes aegypti*. FTIR analysis of the methanolic extract identified functional groups such as aldehyde, amide, alkyl and carbonyl. GC–MS analysis revealed five major components: ursolic acid methyl ester (12.42%), lupeol (53.86%), lupeol acetate (10.95%), cycloeucalenol (2.62%) and hentriacontane (2.64%). These findings indicate that *A. scholaris* flower extracts in different solvents show potential larvicidal and mosquito repellent activity for the control of malaria and dengue.

## 1. Introduction

Mosquito‐borne diseases represent a major global health concern, affecting nearly 700 million people annually. *Aedes aegypti* and *Anopheles stephensi*, primary vectors for dengue and malaria, respectively, pose particular challenges in Asia, Africa and South America. In Pakistan, approximately 60% of the population resides in malaria‐endemic regions, with infants, children and pregnant women being especially vulnerable [1]. Dengue incidence has increased markedly since the 1990s, and Karachi has experienced annual outbreaks since 2005 [2]. The World Health Organization (WHO) currently lists dengue among the top 10 global health threats. To address these tests, there is a need to develop ecologically safe and sustainable mosquito control strategies.

Although synthetic insecticides have traditionally served as the primary method of mosquito control, their drawbacks, including environmental damage, nontarget effects and increasing insect resistance, have become significant concerns. Consequently, there is growing interest in plant‐based alternatives. Many secondary metabolites, which are produced by the plants, can disrupt mosquito biology through various mechanisms. Researchers are therefore increasingly investigating these natural compounds as potential sources of novel larvicidal, repellent and adulticidal agents for mosquito management [3].

Several studies have demonstrated the mosquitocidal potential of remedial plants, including volatile oils. Senthil‐Nathan [4] reviewed multiple plant species exhibiting insecticidal activity against mosquito vectors, including species effective against *Aedes aegypti*, *Aedes albopictus*, *Culex quinquefasciatus* and *Anopheles stephensi*. Essential oils from plants in the Zingiberaceae family have shown promising larvicidal activity against *Aedes* mosquitoes [5]. Similarly, Kamaraj et al. [6] reported the mosquitocidal activity of *Actiniopteris radiata*, while *Acacia nilotica* exhibited strong larvicidal properties [7, 8]. Chellappandian and coworkers [9–11] further highlighted the pesticidal, larvicidal, repellent and adulticidal effects of essential oils against dengue vectors. Additionally, volatile oils from Piper betle leaves demonstrated significant larvicidal and oviposition‐deterrent effects against *Aedes aegypti* [12]. The extensive research on plant‐based mosquito control, *Alstonia scholaris*, particularly its flowers, remains relatively underexplored. This tropical tree, distributed across Africa and Asia, exhibits considerable phytochemical diversity, with over 400 compounds identified, including indole, quinoline, terpenoid and flavonoid alkaloids [13–16]. Traditionally, *Alstonia scholaris* has been used to treat a variety of ailments, such as malaria, asthma, ulcers and snake bites. Recent studies have confirmed its broad pharmacological activities, including antimicrobial, antioxidant and antiplasmodial effects [17–19].

Despite the recognised medicinal applications of *Alstonia scholaris*, the potential of its flower extracts for mosquito vector control remains largely unexplored, particularly for *Anopheles stephensi* and *Aedes aegypti.* The present study aims to address this gap by evaluating whether *Alstonia scholaris* flowers can provide sustainable and biodegradable solutions for controlling these disease vectors [20–22].

## 2. Materials and Methods

### 2.1. Plant Material Collection and Extraction

The samples (blooming flowers were gathered in early November) of *Alstonia scholaris* (L.) R. Br. were collected from the field of the HEJ Research Institute of Chemical and Biological Centre (ICCBS), Karachi, Pakistan. The plant was identified by a Taxonomist of the Department of Botany, University of Karachi. A voucher sample of the flowers of *Alstonia scholaris* was tagged with voucher number # G.H. 94482. The reference sample was deposited in the herbarium. The flowers were washed and dried for seven days in the shade at room temperature (27^o^C–31^o^C) and then pulverised into powder in a hammer mill. Seventy per cent methanol, ethyl acetate, butanol and aqueous solvents were used for extraction. Until used in investigations, the dry extracts were stored in a deep freezer (−4°C) [23–25].

### 2.2. Extraction and Fractionation

The flower extracts were obtained through the ultrasonic solvent extraction technique. *Alstonia scholaris* flowers (8.6 kg) were obtained in 95% methanol (20 L, 3 times, AR grade, Thailand) for 30 min at room temperature. Filtrates were mixed and rotary evaporated (Biobase, RE 2000 Series) at 45°C to obtain 201 g of crude methanolic extract (Meth‐AS), a yellowish‐brown viscous material, with a 5.02 per cent yield of dry powder. The Meth‐AS was kept in the refrigerator until it was utilised for partitioning. Meth‐AS (92 g) was dissolved in 200 mL distilled water and fractionated three times with 200 mL ethyl acetate. The ethyl acetate (EtA‐AS) layers were separated using a separating funnel and afterwards evaporated at 50°C using a rotary evaporator (EtA‐AS, brown solid, 22.5 g, 24.4% yield of the crude extract). Additionally, the aqueous layer was fractionated with *n*‐butanol (200 mL three times). After separating the mixed *n*‐butanol layer with a separating funnel, it was exposed to evaporation (But‐AS), a yellowish‐brown viscous material, 11.3 g, 12.2% yield of the crude extract. Following rotary evaporation, EtA‐AS and But‐AS were transferred to a desiccator and placed on a water bath (Grant, England, 8 h) and in a desiccator (48 h) to remove any remaining solvents or water. To obtain the aqueous layer, the remaining water layer was kept in the refrigerator (Aqs‐AS, brown‐yellow viscous, 48.5 g, 52.7% yield of the total extract) [23].

### 2.3. Mosquito Rearing

The mosquitoes, *Anopheles stephensi* and *Aedes aegypti* larvae, were collected in October 2019 from natural areas in Karachi, Sindh, using the dipping method and established a mosquito colony at the Department of Zoology, University of Karachi. The larval stages were provided with yeast powder and grown in 2.5‐L marble potteries containing 600 mL of distilled water. Pupae were transported to glass beakers (100 mL) containing 80 mL of distilled water and placed in typical mosquito wooden cages (30 × 30 × 30 cm) for adult emergence. Adults were kept occupied with cotton circles soaked in a 10% honey solution. Female mosquitoes were allowed to feed on rabbit blood every 2 days following emergence. Gravid mosquitoes placed their eggs on filter paper strips in glass cups a few days after having a blood meal. Filter paper strips were used to wrap the eggs. Egg papers were dried overnight and stored under laboratory conditions until needed. For hatching, the egg papers were immersed in dechlorinated water, allowing larvae to emerge. Mosquitoes were reared under controlled environmental conditions at 27 ± 2°C, 70% relative humidity, and a 12‐h light:12‐h dark (L:D) photoperiod.

### 2.4. Larvicidal Test

The WHO [26] protocol was adopted for the larvicidal bioassay. The dried ethyl acetate, butanol and aqueous fractions were reconstituted in the appropriate WHO‐recommended solvent (ethanol) to prepare stock solutions, from which the five test concentrations were prepared for the larvicidal bioassays. To detect mortality, five concentrations of each extract were used. All experiments were carried out with 100 mL of dechlorinated tap water in a 200‐mL plastic container. For each test concentration, 20 healthy fourth‐instar larvae were introduced into each cup containing the test solution, along with the corresponding control. Four replicates were performed per concentration (total 80 larvae per concentration). A total of 80 larvae were applied in four replicates to determine the mortality of each solvent.

Larvicidal bioassays were performed following the WHO [26] protocol with a minor modification, following previously published studies employing the same experimental design [27]. Therefore, 20 larvae/extract/concentration were used.

All plastic containers were incubated in a controlled environment (27°C ± 2°C, RH 70% and a 12–12 light–dark cycle). Only the solvents were administered to the control larvae. Mortality was monitored, and dead larvae were discarded until adult emergence.

Mortality was not observed in control groups exposed to solvent‐only treatments, confirming that larval mortality was due to plant‐derived compounds rather than solvent effects. In accordance with the Abbott formula, a mortality correction is applied, if control mortality ranged from 5% to 20%. A solvent control containing dechlorinated water and the corresponding volume of each extraction solvent was included to exclude solvent‐related mortality, following WHO guidelines.

If control mortality was between 5% and 20%, larval mortality was corrected using Abbott’s formula:

Corrected mortality (%) = (*T*−*C*/100−*C*) × 100, where *T* = observed mortality in the treated group and *C* = mortality in the control group.

If control mortality exceeded 20%, the assay was discarded and repeated.

Lethal concentrations (LC_50_) were estimated using probit analysis with corresponding 95% confidence limits (CLs). The toxicity ranking of extracts was determined based on LC_50_ values, with lower LC_50_ values indicating higher larvicidal potency [27].

### 2.5. Repellent Test

Repellent activity was assessed using standard cage assays (30 × 30 × 30 cm). Extracts were dissolved in ethanol to prepare different concentrations (mg/mL). For the initial repellent screening, a concentration of 1250 ppm (1.25 mg/mL), previously identified as an effective larvicidal concentration as shown in Table 1, was used as a standardised test concentration. This concentration was selected for comparative evaluation of repellent activity and does not imply that larvicidal and repellent efficacies are directly correlated.

**TABLE 1:** Different extracts of *Alstonia scholaris* (flower bud), showing larvicidal activity against 4th‐instar larvae of *Ae*. *aegypti L.*, the dengue vector mosquito.

S. no.	Sample	Dose (ppm)	Mortality (%) after 24 h	LC_50_ (95%)	*χ* ^2^	df
1	Meth‐AS	0.0	0.0%	960 (820–1125)	0.84	3
		625	10%			
		800	30%			
		960	50%			
		1120	70%			
		1250	90%			

2	EtA‐AS	0.0	0.0%	1250 (1020–1525)	1.12	3
		312	10%			
		625	30%			
		1250	50%			
		2500	70%			
		3750	90%			

3	But‐AS	0.0	0.0%	2500 (2050–3050)	0.98	3
		625	10%			
		1250	30%			
		2500	50%			
		3750	70%			
		5000	90%			

4	Aqs‐AS	0.0	0.0%	3750 (3200–4400)	1.35	3
		1250	10%			
		2500	30%			
		3750	50%			
		5000	70%			
		6250	90%			

1 mL of each concentration was uniformly applied to a 5‐ to 6‐cm^2^ area of the ventral forearm of human volunteers, and the solution was allowed to dry before exposure. The dose used was 1250 ppm = 1250 mg/L = 1.25 mg/mL.

Five‐ to 7‐day‐old, starved female mosquitoes (*Aedes aegypti*) were used. Treated arms were exposed to approximately 20 mosquitoes per cage for 0–13 h. 15% DEET (N,N‐diethyl‐meta‐toluamide; Johnson Wax Egypt) served as a positive control, while ethanol served as a negative control [28, 29]. Repellency was recorded at 0 h (immediately after application) and at hourly intervals thereafter. Repellency percentage was calculated as follows:

Repellency (%) = (*C*−*T*/*C*) × 100, where *C* = number of mosquito bites in the control and *T* = number of mosquito bites in the test.

All experiments were performed in triplicate.

### 2.6. Statistical Analysis

Mortality data from the larvicidal bioassays were corrected using Abbott’s formula [30] when control mortality ranged from 5% to 20%. Probit regression analysis was performed using POLO‐PC software [31] to estimate the median LC_50_, median repellency time (retention time [RT_50_]), corresponding 95% CLs, slope ± standard error and chi‐square (*χ* ^2^) values. The relative larvicidal potency of the extracts was compared based on LC_50_ values, with lower LC_50_ values indicating greater toxicity [32]. The relative repellent potency of the extracts was compared based on RT_50_ values, with higher RT_50_ values indicating longer lasting repellent activity.

### 2.7. Characterisation of Active Methanolic Extract

#### 2.7.1. Fourier Transform Infrared (FTIR)

10 mg of the dried extract powder was placed in the FTIR spectrometer (Thermo Scientific) (Model: Nicolet iS5), and the spectrum was obtained in the range of 400–4000 cm^−1^ with a resolution of 4 cm^−1^ [33, 34].

#### 2.7.2. Gas Chromatography–Mass Spectrometry (GC–MS)

The bioactive methanolic extract (Meth‐AS) was subjected to GC–MS analysis using an Agilent 7890 A gas chromatograph coupled with an Agilent 7000 A Triple Quadrupole Mass Spectrometer (Agilent Technologies, Santa Clara, CA, USA). An aliquot of 1.5 μL of the extract was injected into the GC–MS system, and the injector temperature was maintained at a maximum of 360°C. The total chromatographic run time was 83.714 min. The mass spectra of the detected compounds were compared with those in the NIST mass spectral library for compound identification.

## 3. Results

The effects of the crude methanolic extract and its ethyl acetate, *n*‐butanol and aqueous fractions from *Alstonia scholaris* flower buds were evaluated against fourth‐instar larvae of *Anopheles stephensi* and *Aedes aegypti*, the primary vectors of malaria and dengue, respectively. Additionally, the repellent activity of the crude methanolic extract and its derived fractions was assessed against adult *Aedes aegypti*. The ethyl acetate, *n*‐butanol and aqueous fractions were obtained through sequential solvent partitioning of the crude methanolic extract, as described in the Methodology section.

### 3.1. Larvicidal Effect

The larvicidal activity of the crude methanolic extract and its ethyl acetate, *n*‐butanol and aqueous fractions of *Alstonia scholaris* flower buds was evaluated against fourth‐instar larvae of *Anopheles stephensi* and *Aedes aegypti*. Larval mortality increased in a concentration‐dependent manner for all tested samples. Among the tested fractions, the crude methanolic extract exhibited the highest larvicidal activity, with LC_50_ values of 312 ppm against *Anopheles stephensi* and 960 ppm against *Aedes aegypti.* The ethyl acetate fraction showed the next highest activity, followed by the *n*‐butanol and aqueous fractions. Overall, *Anopheles stephensi* larvae were more susceptible to the extracts than *Aedes* aegypti, indicating species‐specific differences in sensitivity.

**FIGURE 1:**
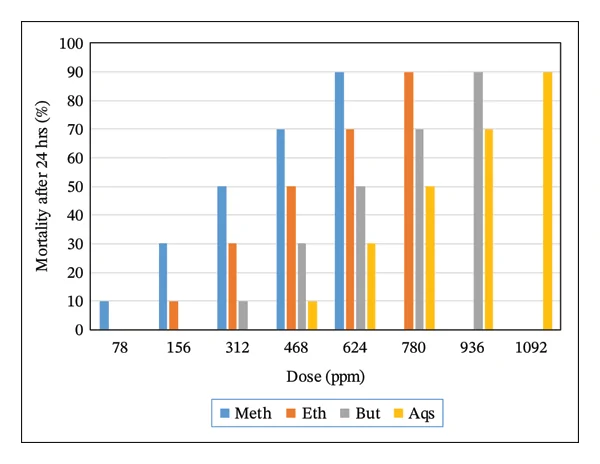
Comparative larvicidal activity of different solvent extracts of *Alstonia scholaris* (flower bud) shows larvicidal activity at different concentrations against 4th‐instar larvae of *Anopheles stephensi,* the main vector mosquito of malaria.

**FIGURE 2:**
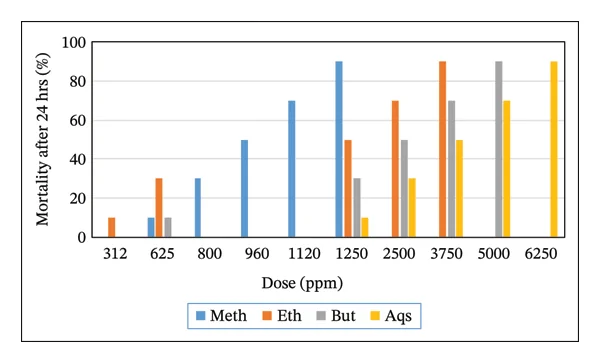
Comparative larvicidal activity of different solvent extracts of *Alstonia scholaris* (flower bud), showing larvicidal activity against 4th‐instar larvae of *Aedes aegypti* L., the dengue vector mosquito.

The larvicidal activity against 4th‐instar larvae of *Anopheles stephensi,* the malaria vector mosquito, was recorded at various concentrations, along with the LC_50_ values reported in Table 2, which are depicted in Figure 1. The statistical analysis is presented in Table 3. In addition, the larvicidal activity against 4th‐instar larvae of *Aedes aegypti,* the dengue vector mosquito, was recorded at various concentrations, along with the LC_50_ values reported in Table 1, which are depicted in Figure 2. The statistical analysis is presented in Table 4. The maximum larval mortality percentage for Meth‐AS (90.0%) occurred at the highest concentration (1280 ppm), whereas the LC_50_ occurred at 960 ppm. The LC_50_ values for EtA‐AS, But‐AS and Aqs‐AS were 1250, 2500 and 3750 ppm, respectively. Simultaneously, the data showed that larval mortality increased with increasing doses of each extract; for example, EtA‐AS was 100% toxic at 3750 ppm, while Aqs‐AS showed only 50% lethality at this dose. However, based on LC_50_ values, the toxicity percentages of *Alstonia scholaris* extracts tested against dengue vectors can be ranked in descending order of larval mortality as follows: Meth‐AS > EtA‐AS > But‐AS > Aqs‐AS.

**TABLE 2:** Different extracts of *Alstonia scholaris* (flower bud), showing larvicidal activity against 4th‐instar larvae of *Anopheles stephensi*, the main vector mosquito of malaria.

S. no.	Sample	Dose (ppm)	Mortality (%) after 24 h	LC_50_ (95%)	*χ* ^2^	df
1	Meth‐AS	0.0	0.0	312 (250–374)	0.00	3
		78	10			
		156	30			
		312	50			
		468	70			
		624	90			

2	EtA‐AS	0.0	0.0	468 (406–530)	0.00	3
		156	10			
		312	30			
		468	50			
		624	70			
		780	90			

3	But‐AS	0.0	0.0	624 (562–686)	0.00	3
		312	10			
		468	30			
		624	50			
		780	70			
		936	90			

4	Aqs‐AS	0.0	0.0	780 (718–842)	0.00	3
		468	10			
		624	30			
		780	50			
		936	70			
		1092	90			

**TABLE 3:** Statistical analysis (probit regression analysis) of different extracts of *Alstonia scholaris* (flower bud), showing larvicidal activity against 4th‐instar larvae of *Anopheles stephensi,* the main vector mosquito of malaria.

Sample	LC_50_	Slope	Intercept
Meth‐AS	312	2.614	−1.297
EtA‐AS	468	3.458	−4.022
But‐AS	624	5.127	−9.174
Aqs‐AS	780	6.675	−14.180

**TABLE 4:** Statistical analysis (probit regression analysis) of different extracts of *Alstonia scholaris* (flower bud), showing larvicidal activity against 4th‐instar larvae of *Ae. aegypti L.*, the dengue vector mosquito.

Sample	LC_50_	Slope	Intercept
Meth‐AS	960	3.271	−5.362
EtA‐AS	1250	3.280	−5.391
But‐AS	2500	3.302	−5.410
Aqs‐AS	3750	3.315	−5.443

The results demonstrated a clear dose‐dependent relationship, with higher extract concentrations resulting in greater mosquito larval mortality after 24 h. Meth‐AS exhibited the greatest lethality, as indicated by the lowest LC_50_ value, while Aqs‐AS was the least toxic, with the highest LC_50_ value in the following order: Meth‐AS > EtA‐AS > But‐AS > Aqs‐AS extracts.

### 3.2. Repellent Effect

**FIGURE 3:**
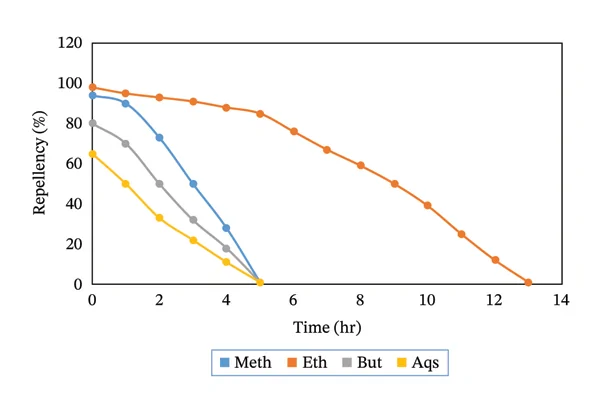
Comparative repellent effect of different solvent extracts of *Alstonia scholaris* (flower bud), showing the repellent activity against adult mosquitoes of *Ae. aegypti* L., the dengue vector.

The repellency (RT_50_) presented in Table 5 of various *Alstonia scholaris* extracts against adult *Aedes aegypti* L., the dengue vector. Over a 9‐h posttreatment period, the EtA‐AS extract from *Alstonia scholaris* maintained RT_50_ (50%) repellency. In comparison, the other three extracts achieved RT_50_ within 3 h after treatment. Specifically, the RT_50_ values for Meth‐AS and But‐AS extracts were 3 and 2 h, respectively. The results also demonstrated a decline in relative repellency over time (Figure 3). The repellent efficacy against fasting *Aedes aegypti* females varied according to the extract. Notably, EtA‐AS provided 9 h of repellency against *Aedes aegypti*, as illustrated in Figure 3. The Probit analysis shown in Table 6 for various *Alstonia scholaris* (flower bud) extracts demonstrates their repellent activity against adult *Aedes aegypti* L., the dengue vector. The repellent effect showed in the following order: EtA‐AS > Meth‐AS > But‐AS > Aqs‐AS.

**TABLE 5:** Different extracts of *Alstonia scholaris* (flower bud), showing the repellent activity against adult mosquitoes of *Ae. aegypti L.*, the dengue vector.

S. no.	Sample code	Time (hours)	Repellency (%)	Biting (%)	RT_50_ (hrs)
1.	Meth‐AS	0	94	06	3
		1	90	10	
		2	73	27	
		3	50	50	
		4	28	72	
		5	01	99	

2.	EtA‐AS	0	100	0	9
		1	95	5	
		2	93	7	
		3	91	9	
		4	88	12	
		5	85	15	
		6	76	24	
		7	67	33	
		8	59	41	
		9	50	50	
		10	39	61	
		11	25	75	
		12	12	88	
		13	01	99	

3.	But‐AS	0	80	20	2
		1	70	30	
		2	50	50	
		3	32	68	
		4	18	82	
		5	01	99	

4.	Aqs‐AS	0	65	35	1
		1	50	50	
		2	30	70	
		3	22	78	
		4	11	86	
		5	01	99	

5.	Ethanol (negative control)	0–5	0	100	0

6.	DEET 15% (positive control)	0	100	0	5
		1	98	2	
		2	92	8	
		3	80	20	
		4	62	38	
		5	50	50	

**TABLE 6:** Probit analysis of different extracts of *Alstonia scholaris* (flower bud), showing the repellent activity against adult mosquitoes of *Ae. aegypti L.*, the dengue vector.

Probit analysis					
Sample	Slope	RT_50_ (h)	95% CI		*χ* ^2^
			Lower	Higher	
Meth‐AS	−3.89	2.84	−4.99 to	−2.78	2.13
EtA‐AS	−1.53	8.18	−1.79	−1.27	18.51
But‐AS	−3.26	1.99	−3.69	−2.83	3.33
Aqs‐AS	−2.60	0.90	−3.13	−2.07	20.83

### 3.3. FTIR Spectroscopy

**FIGURE 4:**
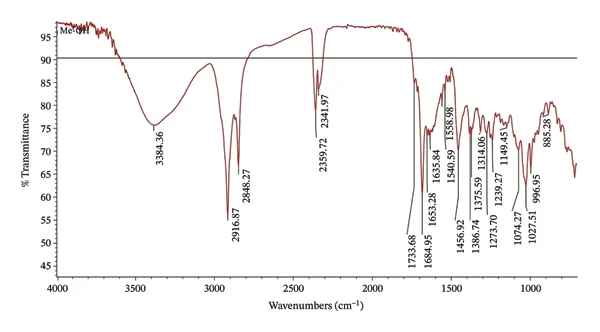
FTIR spectroscopic micrograph representing the functional groups responsible for the presence of phytochemicals using the methanolic extract of *A. scholaris* flower.

The FTIR spectrum (Figure 4) displayed several characteristic absorption bands corresponding to distinct functional groups. A broad absorption peak at 3384 cm^−1^ was attributed to O‐H and/or N‐H stretching vibrations. Peaks at 2916 and 2848 cm^−1^ corresponded to aliphatic C‐H stretching vibrations. Absorption bands at 1733 and 1684 cm^−1^ were assigned to C=O stretching vibrations, while the band near 1630 cm^−1^ indicated C=C and/or C‐N stretching vibrations. The peak at 2359 cm^−1^ may be associated with atmospheric CO_2_ absorption. Bands at 1456, 1386, 1239, 1074 and 996 cm^−1^ corresponded to bending and stretching vibrations of C‐H, C‐O and related functional groups. These findings indicate the presence of hydroxyl, carbonyl and aliphatic functional groups in the extract, as shown in Table 7. While previous studies have reported FTIR analyses of crude *Alstonia scholaris* stem extracts [33, 34], this is the first study to examine the flower.

**TABLE 7:** FTIR spectral analysis of active methanolic extract (Meth‐AS).

Active extracts of flower	Absorption frequency (cm^−1^)	Types of absorption	Interference
Meth‐AS	3384	Medium, broad, bending	Amide groups (N‐H)
	2916	Strong, sharp, stretching	Aldehydes (CHO)
	2848	Sharp, stretching	Alkyl group (C‐H)
	2359	Medium, sharp, stretching	Alkyl group (Alkynes are present)
	1733	Weak, stretching	Carbonyl group
	1684	Medium, sharp, stretching	Amides are present
	1456	Weak, stretching	Carbonyl group (protein)
	1386	Weak, stretching	Anhydrides (C‐O)
	1239	Weak, stretching	Halides (C‐F)
	1074	Weak, stretching	Phenols may be present
	996	Weak, stretching	Anhydrides (C‐O)

**FIGURE 5:**
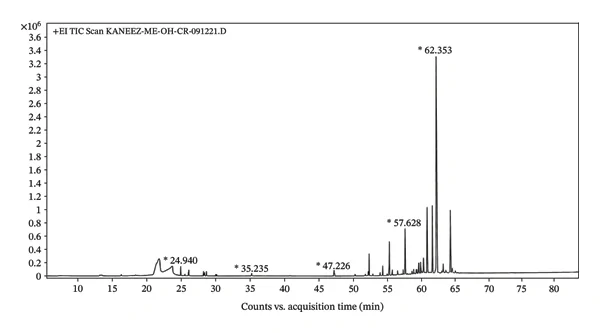
Chromatogram of active methanolic extract obtained by GC–MS.

**FIGURE 6:**
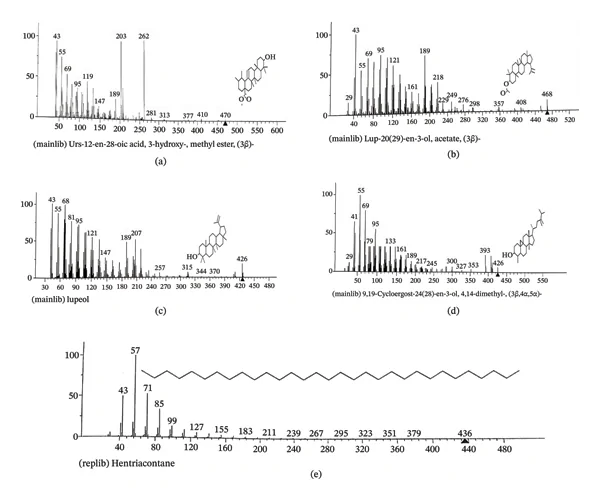
Spectra of chemical constituents presenting with structures obtained in GC–MS. (a) Ursolic acid methyl ester, (b) lupeol acetate, (c) lupeol, (d) cycloeucalenol, and (e) hentriacontane.

GC–MS analysis (Figure 5) revealed 27 peaks between 64.331 and 55.333 RT, with five major components: ursolic acid methyl ester, lupeol acetate, lupeol, cycloeucalenol and hentriacontane. These structures are shown in Figure 6. The methanolic extract of *Alstonia scholaris* contained several bioactive phytoconstituents, identified via the NIST mass spectral library. Their identities were confirmed using RT, molecular formula, molecular weight, major fragment ions (m/z) and similarity index (SI)/match factor, as detailed in Table 8.

**TABLE 8:** Chemical constituents of the methanolic extract of the *Alstonia scholaris* flower obtained by GC–MS.

Sample	RT	% area sum	Chemical constituent	Molecular formula	Molecular weight (g/mol)	Major fragment ions (m/z)	SI/match factor (NIST)
Meth‐AS	64.335	12.42	Ursolic acid methyl ester	C_31_H_50_O_3_	470.73	470, 455, 262, 203, 189	93
	62.353	53.86	Lupeol acetate	C_32_H_52_O_2_	468.75	468, 408, 249, 218, 189	95
	60.885	10.95	Lupeol	C_30_H_50_O	426.72	426, 411, 393, 218, 207, 189	94
	60.333	2.62	Cycloeucalenol	C_30_H_50_O	426.72	426, 411, 408, 393, 300 285,	91
	57.628	2.64	Hentriacontane	C_31_H_64_	436.84	436, 309, 295, 281, 57	90

## 4. Discussion

Recent studies have been conducted to replace synthetic pesticides with natural organic pesticides to reduce environmental hazards and improve public health. These alternatives can be used as repellents or eradicators to reduce, destroy or kill pests. Previous studies have identified neem, lavender and cottonseed oils as the most effective natural pesticides with long‐lasting activity. Similarly, mint and garlic oils showed strong repellency against various insects, with long‐lasting effects [35]. Oguh et al. [36] reported that several natural pesticides, such as rotenone (Derris sp.), nicotine, carboxin, fluoroacetate, neem (*Azadirachta indica*), microbial pesticide pyrethrins and *Bacillus thuringiensis*, usually target insects at their targeted sites of action, such as the nervous system, resulting in knockdown, lack of coordination, paralysis and death [37].

This study found that the methanolic extract of *Alstonia scholaris* flower buds had notable larvicidal activity against *Anopheles stephensi* and *Aedes aegypti* larvae, whereas the ethyl acetate extract was a stronger repellent. The LC_50_ values here were higher (lower potency) than those reported in prior studies using other plant parts or extraction systems. These differences likely result from variations in plant part, extraction solvent, phytochemicals, geographic origin, climate and mosquito species.

Kaushik and Saini [15] reported the larvicidal activity of the acetone extract of *Alstonia scholaris* leaves (LC_50_ 239.9 ppm) against fourth‐instar larvae of *Aedes aegypti*. A comparative study can be made with the study of Kaushik and Saini, who reported strong larvicidal activity of the acetone extract of *Alstonia scholaris* leaves against fourth‐instar *Aedes aegypti* larvae with an LC_50_ value of 239.9 ppm. In contrast, the methanolic flower extract used in the present study showed comparatively higher LC_50_ values against both *Anopheles stephensi* and *Aedes aegypti*. This variation may primarily be due to differences in the plant part analysed. Leaves are often richer in defensive secondary metabolites such as alkaloids, phenolics and terpenoids because they are directly exposed to herbivores and environmental stress, whereas flower buds may contain comparatively lower concentrations of these bioactive compounds. In addition, acetone and methanol differ in polarity and extraction efficiency, which can significantly influence the types and quantities of phytochemicals extracted [37]. Therefore, the stronger activity reported by Kaushik and Saini may be associated with the solvent’s ability to extract more potent larvicidal constituents from leaves.

Geographical and environmental factors also affect biological activity. Phytochemical content in plants varies with climate, soil, altitude, season and location, altering active metabolite concentrations. Differences in mosquito strain, larval stage, exposure time and experimental conditions may also influence LC_50_ values and outcomes [38].

The latest study also reported the synthesis of Fe_2_MgO_4_ nanoparticles (NPs) using leaf extracts of *Alstonia scholaris* and Polyalthia longifolia. The prepared NPs were assessed for their larvicidal activity. The NPs were potent against *Aedes albopictus* larvae, with LD_50_ values of 5.61 and 9.13 μg/mL for the AS‐ and PL‐synthesised Fe_2_MgO_4_ NPs, respectively [39]. Yadav et al. [40] reported larvicidal activity of methanolic leaf extracts of *Alstonia scholaris* against *Aedes albopictus* larvae with an LC_50_ value of 473.19 ppm, supporting the moderate but significant larvicidal efficacy observed in the current study. Similarly, NP formulations synthesised using *Alstonia scholaris* leaf extracts exhibited enhanced larvicidal activity against *Aedes larvae*, suggesting that formulation strategies may further improve the bioefficacy of plant‐derived compounds [41, 42].

In contrast, biopesticides are considered safer than synthetic insecticides because they degrade rapidly in the environment and can reduce negative environmental impacts. Therefore, different plant extracts have been investigated for use against public health pests, such as *Foeniculum vulgare, Azadirachta indica* and *Tribulus terrestris*. Nathan et al. [43] found that essential oil from *Eucalyptus tereticornis* had larvicidal action against *Anopheles stephensi* larvae with LC_50_ and LC_90_ values of 23.8 and 63.9 ppm, respectively.

Mosquitoes have developed resistance to many synthetic pesticides, requiring natural alternatives. Crude solvent extracts, essential oils and fractions from native plants could serve as potential reservoirs [44]. Mosquito–human interactions are linked to a slew of diseases. Mosquitoes transmit malaria, dengue fever, chikungunya fever, yellow fever and other serious and well‐known diseases. In humans and livestock worldwide, these illnesses cause major morbidity and mortality. Furthermore, underdeveloped countries such as Pakistan, where the frequency of malaria and dengue fever is still high, and treatment capacity is limited [45]. Therefore, greater focus should be placed on implementing and controlling vector‐borne disease prevention measures. *Alstonia scholaris* has been found to be environmentally safe, nontoxic to humans, and to possess a wide range of medicinal properties [46, 47].

The findings of this study on *Aedes aegypti* larval mortality were consistent with those reported by El‐Sheikh et al. [48], Maurya et al. [45], Govnidarajan [49], Patil et al. [50] and Kovendan et al. [47]. The acetone extract of *Alstonia scholaris* Linn (Apocynaceae) was tested for larvicidal efficacy against the mosquito vector *Aedes aegypti* [35]. For 3^rd^‐instar larval *Aedes aegypti*, the LC_50_ value of leaf acetone *Alstonia* extracts was estimated to be 185 parts per million (ppm). Pavela [51] employed essential oils from *Thymus vulgaris*, *Satureja hortensis* and *Thymus satureioides* against *Aedes aegypti* larvae. Sharma et al. [52] employed a petroleum ether extract of *Alstonia annua* against *Anopheles stephensi* and *Culex quinquefasciatus* larvae. The methanol extract had a significant effect on *Anopheles stephensi* for the 4th instar with LC_50_ 312 ppm, and *Aedes aegypti* for the 4th instar, with an LC_50_ of 960 ppm, in the current investigation. Thus, the toxicity of Alstonia plant extract is more against *Anopheles stephensi* compared to *Aedes aegypti*. Similarly, different extracts exhibited different repellencies. In contrast, the ethyl acetate extract had a significant repellency time of 9 h. El‐Sheikh et al. [48] also tested the repellency activity of *Tribulus terrestris* and found a considerable effect. However, the larvicidal and repellent activities of *Alstonia scholaris* flower buds against *Anopheles stephensi* and *Aedes aegypti* for the 4th instar have not yet been identified.

The toxicity of plant extracts against the 4th‐instar larvae of *Anopheles stephensi* and *Aedes aegypti* for dengue and malaria was most significant, with the methanolic extract showing the highest larvicidal activity, according to the findings. However, the ethyl acetate extract exhibited a strong repellent effect against *Aedes aegypti*. Starving female adults of *Aedes aegypti* showed repellency activity at all doses of plant extracts used in this study. The tested repellent activity of plant extracts may vary depending on the solvent and the amount of extract. The ethyl acetate extract of the plant was more successful in repelling the mosquitoes examined than the methanol, butanol and aqueous extracts. Many plant extracts and essential oils have been shown to repel various mosquito species [53]. To investigate insecticidal effect against *Aedes aegypti*, Gunasekaran et al. [54] utilised Patil et al. [50] used *Plumbago zeylanica* (Plumbaginaceae) and *Cestrum nocturnum* (Solanaceae) plant extracts, and Kovendan et al. [47] applied Carica papaya plant extracts (Caricaceae).

The GC–MS analysis of the active methanolic extract revealed the presence of several bioactive phytoconstituents, including ursolic acid methyl ester, lupeol, lupeol acetate, cycloeucalenol and hentriacontane. Among these, lupeol acetate represented the major constituent based on peak area percentage. Previous studies have reported that triterpenoids such as lupeol and lupeol acetate possess insecticidal and larvicidal activities against mosquito vectors [55, 56]. Therefore, the larvicidal and repellent activities observed in this study may be associated with the combined or synergistic effects of these phytochemicals. Therefore, these compounds are considered as potential candidate or bioactive constituents that warrant further investigation through isolation, purification and bioassay‐guided studies to confirm their specific mechanism of action.

Overall, although the flower extracts of *Alstonia scholaris* demonstrated lower potency compared with some leaf‐based extracts reported previously, the present study provides the first systematic evaluation of flower extracts against both *Anopheles stephensi* and *Aedes aegypti* for larvicidal and repellent activity. These findings support the potential of *Alstonia scholaris* as a source of environmentally friendly mosquito control agents and highlight the importance of further studies focusing on the purification of active compounds, optimisation of extraction methods and formulation development to improve efficacy.

In recent years, many researchers have focused on developing effective insecticides and pesticides from natural sources. This is a current trend due to the disadvantages of synthetic chemical pesticides, such as their impact on the atmosphere, the development of resistance mechanisms, the induction of toxicity in nontarget organisms and humans, and the development of resistance in targeted insect populations [57]. The insecticidal properties of *Alstonia scholaris* could be a promising control strategy against mosquito vectors, potentially leading to the development of nontoxic insecticides, especially in developing countries such as Pakistan [58].

The present study demonstrated that the methanolic extract of *Alstonia scholaris* flower buds exhibited notable larvicidal activity against fourth‐instar larvae of *Anopheles stephensi* and *Aedes aegypti*, whereas the ethyl acetate extract showed comparatively stronger repellency activity. Although the observed larvicidal activity was significant, the LC_50_ values obtained in the current study were comparatively higher (indicating lower potency) than those reported in some previous investigations using other plant parts or extraction systems. These differences may be attributed to variations in plant part used, extraction solvent, phytochemical composition, geographical origin, climatic conditions and mosquito species tested.

A direct comparison can be made with the study of Kaushik and Saini [15], who reported strong larvicidal activity of the acetone extract of *Alstonia scholaris* leaves against fourth‐instar *Aedes aegypti* larvae with an LC_50_ value of 239.9 ppm. In contrast, the methanolic flower extract used in the present study showed comparatively higher LC_50_ values against both *Anopheles stephensi* and *Aedes aegypti.* This variation may primarily be due to differences in the plant part analysed. Leaves are often richer in defensive secondary metabolites such as alkaloids, phenolics and terpenoids because they are directly exposed to herbivores and environmental stress, whereas flower buds may contain comparatively lower concentrations of these bioactive compounds. In addition, acetone and methanol differ in polarity and extraction efficiency, which can significantly influence the types and quantities of phytochemicals extracted. Therefore, the stronger activity reported by Kaushik and Saini may be associated with the solvent’s ability to extract more potent larvicidal constituents from leaves.

Spatial distribution and environmental factors may also contribute to differences in biological activity. The phytochemical composition of medicinal plants varies with climate, soil conditions, altitude, seasonal variation and geographical location as mentioned above. Such variations can alter the concentration of active metabolites responsible for mosquitocidal activity. Furthermore, differences in mosquito strain susceptibility, larval stage, exposure duration and experimental conditions among studies may additionally influence LC_50_ values and biological responses [59].

The findings of the present investigation are nevertheless comparable with previous reports demonstrating the mosquitocidal potential of *Alstonia scholaris*. Yadav et al. [40] reported larvicidal activity of methanolic leaf extracts of *Alstonia scholaris* against *Aedes albopictus* larvae with an LC_50_ value of 473.19 ppm, supporting the moderate but significant larvicidal efficacy observed in the current study. Similarly, NP formulations synthesised using *Alstonia scholaris* leaf extracts exhibited enhanced larvicidal activity against *Aedes larvae,* suggesting that formulation strategies may further improve the bioefficacy of plant‐derived compounds [41].

The GC–MS analysis of the active methanolic extract revealed the presence of several bioactive phytoconstituents, including ursolic acid methyl ester, lupeol, lupeol acetate, cycloeucalenol and hentriacontane. Among these, lupeol acetate represented the major constituent based on peak area percentage. Previous studies have reported that triterpenoids such as lupeol and lupeol acetate possess insecticidal and larvicidal activities against mosquito vectors [55, 56]. Therefore, the larvicidal and repellent activities observed in this study may be associated with the combined or synergistic effects of these phytochemicals.

Overall, although the flower extracts of *Alstonia scholaris* demonstrated lower potency compared with some leaf‐based extracts reported previously, the present study provides the first evidence of larvicidal and repellent activities of *Alstonia scholaris* flower bud extracts against both *Anopheles stephensi* and *Aedes aegypti.* These findings support the potential of *Alstonia scholaris* as a source of environmentally friendly mosquito control agents and highlight the importance of further studies focusing on the purification of active compounds, optimisation of extraction methods and formulation development to improve efficacy.

## 5. Conclusion

The study demonstrated that the flower extract of *Alstonia scholaris* exhibits significant larvicidal and repellent effects against *Anopheles stephensi* and *Aedes aegypti*. The primary constituents, identified by the highest area sums in the GC–MS analysis, can serve as active ingredients in formulations. Additionally, the active fraction can be isolated and purified to obtain bioactive compounds. Consequently, this plant material serves as an effective larvicidal and repellent agent. Kallur and Patil [60] also assessed *Alstonia scholaris* against the pest *Rhyzopertha dominica*, revealing considerable pesticidal activity.

### 5.1. Limitations of the Study

The present study provides preliminary evidence regarding the larvicidal and repellent potential of *Alstonia scholaris* flower extracts against *Anopheles stephensi* and *Aedes aegypti*. However, several limitations should be acknowledged. First, the LC_50_ values obtained in this study were comparatively higher than those reported for some synthetic insecticides and certain plant‐derived extracts, indicating relatively lower potency of the crude flower extracts. Therefore, although the extracts demonstrated biological activity, further optimisation and purification may be required before practical field application.

Second, the GC–MS analysis identified several phytochemical constituents; however, the active principles responsible for the observed larvicidal and repellent effects were not isolated or individually evaluated. Consequently, the observed biological activities cannot be directly attributed to any single compound, and further bioassay‐guided fractionation and mechanistic studies are required to identify the specific active constituents and their modes of action.

Experimental retention indices (RIs) were not determined because a homologous *n*‐alkane standard mixture was not analysed under the same GC–MS conditions. Therefore, compound identification was considered tentative and was based on NIST mass spectral library matching, characteristic fragmentation patterns and comparison with published literature.

Third, the use of a single standardised concentration for human volunteer repellent assays. Future studies should determine effective repellent concentrations using laboratory behavioural assays (e.g., Y‐tube olfactometer) before human testing.

Fourth, the repellency assay conducted in the present study was preliminary and involved application to a limited skin surface area under controlled laboratory conditions. Such assays may not fully represent real‐world environmental exposure or long‐term protection efficacy. Therefore, additional large‐scale studies involving standardised field conditions, formulation stability, safety evaluation and dermal toxicity assessment are necessary before recommending practical use in humans. Another limitation relates to possible variability in plant phytochemical composition. The concentration and composition of secondary metabolites in medicinal plants can vary significantly depending on geographical location, climatic conditions, soil composition, seasonal variation, harvesting period and extraction methods. Such variability may affect the reproducibility and consistency of larvicidal and repellent activity across studies.

Despite these limitations, the present work provides important baseline data supporting the potential of *A. scholaris* flower extracts as environmentally friendly mosquito control agents and establishes a foundation for future phytochemical and formulation‐based investigations.

## Funding

No funding has been obtained for the particular research.

## Ethics Statement

The repellent bioassay involving human volunteers was conducted in accordance with institutional ethical guidelines. Ethical approval was obtained from the Ethics Committee of the Board of Advanced Studies and Research, BASR, Baqai Medical University, under approval number Ref: BMU EC/04‐2019‐02. Transcribed informed consent was acquired from all volunteers earlier to participation.

## Conflicts of Interest

The authors declare no conflicts of interest.

## Data Availability

The data supporting the findings of this study are available from the corresponding author upon reasonable request.
